# Polyphenol Profile and Antioxidant, Antityrosinase, and Anti-Melanogenesis Activities of Ethanol Extract of Bee Pollen

**DOI:** 10.3390/ph17121634

**Published:** 2024-12-05

**Authors:** Qiang He, Jie Wang, Jingjing Li, Wenchao Yang

**Affiliations:** College of Bee Science and Biomedicine, Fujian Agriculture and Forestry University, Fuzhou 350002, China; h3077ang@163.com (Q.H.); wangjie01092023@163.com (J.W.); lijingjing000407@163.com (J.L.)

**Keywords:** polyphenols, antioxidant, antityrosinase, anti-melanogenesis, bee pollen

## Abstract

**Background/Objective:** Bee pollen, a rich nutritional food, was employed to develop a raw material for skin whitening. **Methods:** The polyphenol profile and antioxidant, antityrosinase, and anti-melanogenesis activities of the ethanol extracts of five species of bee pollens (EEBPs) were determined. **Results:** The results showed that there were a total of 121 phenolic compounds in these EEBPs. Each type of bee pollen had unique substances. The best anti-melanogenesis activity was observed for sunflower EEBP, about 25% at a concentration of 25 μg/mL BEEP. The anti-melanogenesis activities of EEBPs from high to low were sunflower, apricot, camellia, rapeseed, and lotus EEBPs. The anti-melanogenesis activity in B16F10 cells was positively correlated with the antityrosinase activity and total phenol content, with coefficients of 0.987 and 0.940. The Kyoto Encyclopedia of Genes and Genomes enrichment analysis results of untargeted proteomics revealed that sunflower EEBP inhibited melanogenesis in B16F10 cells by reducing the expression of the proteins MAP2K1, NFKB2, RELB, RPS6KA3, CASP3, TRAF6, MAP2K5, MAPKAPK3, STRADA, CCNA2, and FASN involved in the cAMP, MAPK, and TNF signaling pathways, even though these pathways were not significantly different from the control group. **Conclusions:** The sunflower EEBP has high inhibition effect on melanogenesis than other species EEBPs. The results provide a basis for the future industrial development of a raw material for skin whitening.

## 1. Introduction

Bee pollen is composed of copious amounts of pollen grains, which were foraged by workers using the weak electrostatic field generated between the negatively charged flower and the positively charged body, mixed with a small dose of the secretions from salivary glands or nectar, and placed in specific baskets situated on the tibia of their hind legs [1,2]. There are many biological substances, including carbohydrates, proteins, vitamins, minerals, polyphenols, dietary fiber, volatile compounds, and lipids, in bee pollen. The moisture content ranges from 1.82 to 7.33% in commercial bee pollen [3] and 20 to 30% in the original bee pollen [4]. Bee pollen contains approximately 40–85% carbohydrates, including 15.2–22.4% fructose, 7.0–21.9% glucose, 14–19.8% sucrose, and others (W/W, dry weight, DW) [5]. There are approximately 13.6–26.8% (W/W, DW) protein with a total of 20 amino acids, 1–10% total lipids, 0.0019–0.0344% vitamin B, 0.0015% vitamin A, 0.0062% vitamin E (α-tocopherol), and trace amounts of vitamin C in bee pollen [5,6]. It contains rich micronutrients such as potassium (K), sodium (Na), calcium (Ca), magnesium (Mg), phosphorus (P), zinc (Zn), iron (Fe), copper (Cu), and manganese (Mn) [5]. There is also a very diverse group of yellow-to-red-color polyenes, polyphenols, dietary fiber, and volatile compounds, among which polyphenols include 19 phenolic acids, 25 flavonoids, and seven glycosylated flavonoids [3,4,5,6,7,8]. The chemical compositions of diverse bee pollens vary from different botanical origins, geographic origins, entomological origins, processing methods, and storage conditions [2,3,4,5,6,7,8]. These chemical compositions decide the functional effects of bee pollen, such as antioxidant, antifungal, antimicrobial, antiviral, anti-inflammatory, hepatoprotective, anticancer, immunostimulatory, radical-scavenging, gut-microbiota-modulating, and local analgesic activities [5,9,10,11].

The antioxidant capability is one of the significant bioactivities of bee pollen. This primarily depends on the presence of various compounds, including 4-hydroxybenzoic, *p*-coumaric, phenyllactic, caffeic, transcinnamic, syringic, 3,4 dimethoxycinnamic (acid), chlorogenic, rosmarinic, and vanillic acids; apigenin; caffeic acid phenethyl ester (CAPE); chrysin; epicatechin; hesperetin; taxifolin; myricetin; catechin; genistein; isorhamnetin; kaempferol; luteolin; pinocembrin; quercetin; and rutin [8]. Enzymes like catalase and superoxide dismutase (SOD), along with glutathione (GSH), also play roles in the antioxidant effects of bee pollen [12]. The antioxidant capability is influenced by several factors, including the processing method [13,14,15], botanical origin [8,16], storage [17], geographical origin [18], and extraction solvents [14,19].

In addition to the in vitro antioxidant activity, in vivo studies have reported benefits. *Cistaceae* bee pollen modulates the expression of antioxidant enzymes in the liver, brain, and lysate of erythrocytes and reduces hepatic lipid peroxidation levels in mice [20]. The water and methanol extracts of bee pollen increase the SOD level and the total antioxidant capacity and significantly reduce the malondialdehyde (MDA) content in mice and rats [12,21]. Bee pollen relieves the oxidative stress on hepatocytes [22], oxidative damage and reactive oxygen species produced by propionic acid [23,24], and NaF-induced oxidative stress in rats [25]. Furthermore, bee pollen alleviates oxidative stress in hepatocytes and both oxidative damage and reactive oxygen species produced by propionic acid and sodium fluoride in rats [26].

Beyond the antioxidant activity, polyphenols also possess the ability to inhibit tyrosinase [27], which is a biofunctional copper-containing enzyme that plays a crucial role in the melanin biosynthesis pathway within melanocytes. Tyrosinase catalyzes the hydroxylation of monophenols and the oxidation of diphenols to form quinines [28]. Melanin is the primary determinant of skin color and a vital substance to prevent skin damage caused by UV radiation and oxidative stress [29]. However, the excessive accumulation of melanin might result in hyperpigmentation diseases, such as lentigo, ephelides, melasma, freckles and nevus, and melanomas, which may increase the risks of both cancer and Parkinson’s disease [30,31,32,33]. Consequently, tyrosinase inhibitors are being widely studied as treatments for pigmentation issues and for the development of skin-whitening agents [27,28,34,35,36,37,38,39]. Examples of natural compounds with antityrosinase activity include a quercetin-loaded olive oil nanoemulsion [34], flavonoids from *Trifolium nigrescens* subsp. petrisavi [36], citrus essential oils [37], mango seed kernel extract [38], and safflospermidines separated from the bee pollen of *Helianthus annuus* L. [39]. Notably, safflospermidines exhibit higher antityrosinase activity than kojic acid in vitro. Traditional tyrosinase inhibitors, such as kojic acid, aloesin, hydroquinone, and α- and β-arbutins, have been used as skin-whitening agents [27]. However, there is a need for more effective and safer tyrosinase inhibitors, as cytotoxicity or side effects may be caused by irritation, peeling, redness, or stinging in daily life [40].

There are limited research reports on the antityrosinase and antioxidant activities of bee pollen harvested in China. In this paper, the polyphenol profiles and antioxidant, antityrosinase, and anti-melanogenesis activities of sunflower bee pollen extract in B16F10 melanoma cells were determined to discover an effective and safer tyrosinase inhibitor for further utilization as a raw material for skin-whitening agents.

## 2. Results

### 2.1. Components in Ethanol Extracts of Bee Pollens

#### 2.1.1. TPCs and TFCs in Ethanol Extracts of Bee Pollens

There were significant differences among the TPCs and TFCs of ethanol extracts of various species of bee pollen (EEBP) (Table 1). The TPCs and TFCs of bee pollen samples ranged from 5.36 to 132.57 mg GAE/g and 1.99 to 109.06 mg RE/g calculated by the equations of the standard curves y = 0.00567x + 0.07371 (r^2^ = 0.9929) and y = 0.00148x + 0.05319 (r^2^ = 0.9929), respectively. 

#### 2.1.2. The Polyphenols in Ethanol Extracts of Bee Pollens

**Figure 1:**
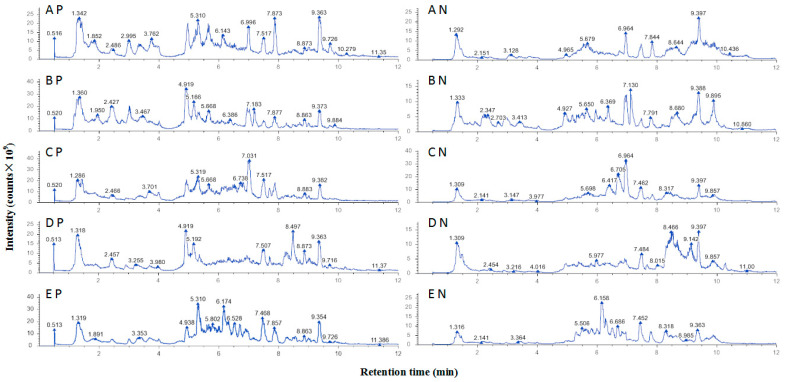
UHPLC–MS/MS ion spectra of ethanol extracts of bee pollens: A, B, C, D, and E represent rapeseed, apricot, camellia, lotus, and sunflower bee pollens, respectively. P and N represent positive ion mode and negative ion mode, respectively.

The ion spectra of ethanol extracts of bee pollens were shown in Figure 1. A diverse variety of polyphenols has been identified in five species of EEBPs. In total, 71 polyphenolic compounds were found, with significant variation across different bee pollen samples (Table 2).

EEBPs of rapeseed, camellia, sunflower, lotus, and apricot bee pollens contain 56, 46, 45, 42, and 35 polyphenolic compounds, respectively. Certain flavonoids are unique to specific types of bee pollen: orientin, (−)-catechin gallate, methyl hesperidin, hesperidin, and epicatechin are found only in rapeseed bee pollen; chloropelargonidin is exclusive to camellia bee pollen; violacein and tricin-O-malonylhexoside are present only in sunflower bee pollen; and heptamethoxyflavonoids, isovitexin, chlorocyanidin, and baohuoside I are unique to lotus bee pollen. In terms of the relative content, camelliaside A is the most abundant flavonoid in rapeseed bee pollen; rutin is the most prevalent in both lotus and apricot bee pollens; kaempferol has the highest relative content in camellia bee pollen; and quercetin-3β-D-glucoside is the dominant flavonoid in sunflower bee pollen.

### 2.2. Antioxidant and Tyrosinase Inhibitory Capacities of Ethanol Extracts of Bee Pollens

The antioxidant capacities of EEBPs are shown in Table 3. The antioxidant capacities of sunflower EEBP were highest among those of the other four species EEBPs determined by the DPPH, FRAP, and ABTS assays.

**Figure 2:**
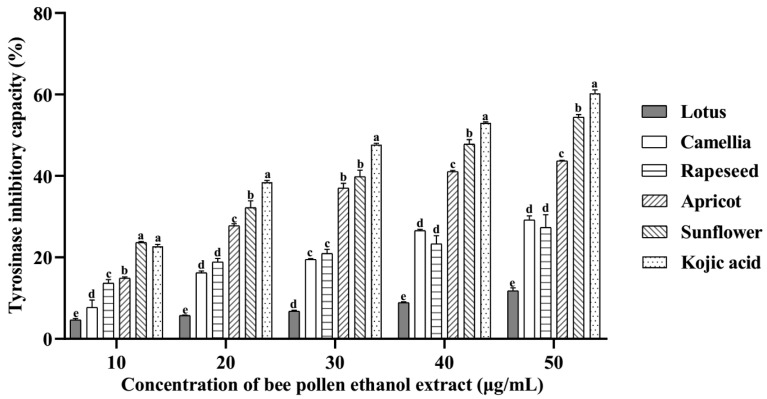
The antityrosinase activities of ethanol extracts of bee pollens. Note: Different lowercase characters above the same concentration mean significant differences (*p* < 0.05).

The tyrosinase inhibitory capacities of EEBP are shown in Figure 2. The five EEBPs inhibited the activity of tyrosinase in a concentration-dependent manner. There were significant differences in the inhibition rate among different species of EEBPs. The sunflower EEBP had much higher inhibition rates for tyrosinase activity than the EEBPs of apricot, rapeseed, camellia, and lotus. At a concentration of 50 μg/mL, the inhibition rate of the sunflower EEBP was higher than 50%, while those of the other EEBPs were lower than 50%.

### 2.3. Anti-Melanogenesis Activity of Ethanol Extracts of Bee Pollens on Melanin Production in Mouse B16F10 Melanoma Cells 

**Figure 3:**
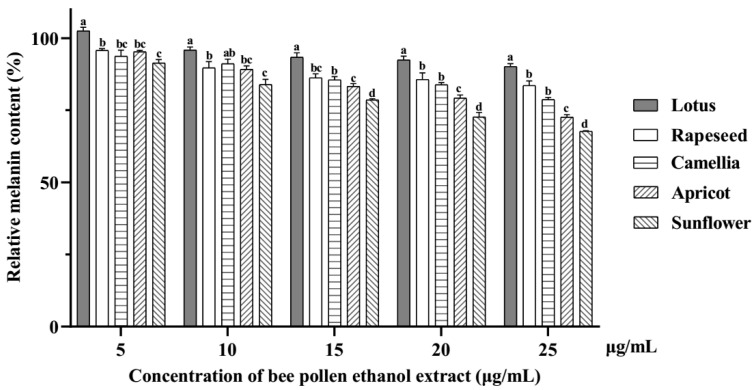
The relative melanin content in B16F10 cells treated with ethanol extracts of bee pollens. Note: Different lowercase characters above the same concentration mean significant differences (*p* < 0.05).

The relative melanin contents in B16F10 cells treated with EEBPs decreased in a concentration-dependent manner (Figure 3). The highest anti-melanogenesis activity was observed for sunflower EEBP, which was about 25% at 25 μg/mL BEEP, followed by apricot, camellia, rapeseed, and lotus EEBPs.

The correlation coefficients among DPPH, FRAP, ABTS, TPC, TFC, tyrosinase inhibitory, and anti-melanogenesis activities showed that the anti-melanogenesis activity was highly dependent on the TPCs in the EEBPs (*p* < 0.05, Table 4).

### 2.4. Anti-Melanogenesis Mechanism of the Ethanol Extract of Sunflower Bee Pollen on Melanin Production in Mouse B16F10 Melanoma Cells 

The DIA quantitative proteomics analysis identified a total of 7555 proteins. Compared to the control group, 272 proteins were differentially expressed, consisting of 171 up-regulated and 101 down-regulated differentially expressed proteins (DEPs). The volcano map of differentially expressed proteins in B16F10 cells (sunflower EEBP vs. control) is shown in Figure S2. Analyzing the subcellular localization of these differentially expressed proteins revealed that 28.35% were located in the cytoplasm, 27.84% in the nucleus, and 11.34% in the mitochondria. The Gene Ontology (GO) enrichment analysis indicated that the differentially expressed proteins primarily participated in metabolic processes, catalytic activities, redox processes, and calcium ion binding. Additionally, the KEGG enrichment analysis suggested that the sunflower EEBP inhibited melanogenesis in B16F10 cells by reducing the expression of proteins associated with the cAMP, MAPK, and TNF signaling pathways (Table 5), which are highly related to melanogenesis, even though these pathways were not significantly different from the control group.

## 3. Discussion

The polyphenol components varied mainly depending on the botanic source. In this experiment, the TPC of bee pollen samples ranged from 5.39 to 132.51 mg GAE/g EEBP. This range is similar to other research if the extraction rate was included, which were 9.41 to 27.49 [41], 4.64 to 17.93 [42], and 12.60 to 84.22 mg GAE/g bee pollen [43], and 15.73 to 26.92 [44], 4.2 to 29.6 GAE mg/g extract of bee pollen [45]. The EEBP with the highest TPC was sunflower bee pollen, which was different from previous reports, which were 12.29 mg GAE/g dried bee pollen from Hungary [41], 7.56 mg GAE/g dried bee pollen from Romania [42], and 2.907 mg GAE/g of dried bee pollen harvested from Serbia [46]. Meanwhile, the EEBP with the lowest TPC was lotus bee pollen, which was similar to the reported values [47] of 1.81 mg PAE/g bee pollen from China extracted with 75% ethanol/water solvents and determined by protocatechuic acid (PA) as the standard solution. The EEBP with a medium TPC was rapeseed, 12.57 mg GAE/g dried bee pollen from China [48] and calculated by free and bound phenolic extracts and measured by the same method as this paper. There was 12.90 mg PAE/g bee pollen from China extracted by 75% ethanol/water solvents [47]. This difference in the TPC may be related to the different geographic origins, extraction solvents, and storage conditions of bee pollen.

The TPC mainly depends on the polyphenols in bee pollen. EEBPs of rapeseed, camellia, sunflower, lotus, and apricot bee pollens contain 56, 46, 45, 42, and 35 polyphenols. Reports showed that there were 37 phenolic compounds in sunflower bee pollen from Serbia [46], 35 phenolic compounds in eight bee pollen samples from Morocco [49], and 258 phenolic compounds in thirty-two bee pollen samples from Italy [44]. The different phenolic compounds in bee pollen depend on the geographic and botanical origins, storage conditions, extraction solvent, and determination methods of bee pollen.

The antioxidant activities of various species of bee pollen are mostly attributed to their polyphenols. Consistency exists in the antioxidant activities from the DPPH, FRAP, and ABTS assays. There is a consistency in the DPPH (IC_50_) and ABTS assays [47]. EEBPs from five of a total of fourteen species from high to low were rapeseed = camellia > apricot > sunflower > lotus [47], which were similar to sunflower > rapeseed > camellia > apricot > lotus in this experiment. This different result was caused by the different expression method, TE per bee pollen or EEBPs. The phenolic compounds can directly participate in antioxidant activity [50]. This is consistent with the results of this study, where the correlation coefficients between the phenolic content and antioxidant activity were higher than 0.858.

Tyrosinase is the key enzyme for melanin production in the human body. Inhibiting tyrosinase can effectively reduce melanin synthesis, thereby achieving a whitening effect on the skin [51]. Some studies have found that phenolamide in bee pollen is a component that affects the tyrosinase activity [52]. In this study, the five EEBPs inhibited tyrosinase in a concentration-dependent manner. The inhibitory activity of sunflower EEBP toward tyrosinase was the highest. Sunflower bee pollen from Thailand also showed inhibition of tyrosinase activity after extraction with methanol [39], which is consistent with the results of this study. However, some studies have shown that apricot bee pollen, after extraction with ethyl acetate, has a better inhibitory effect on tyrosinase activity than sunflower bee pollen [52]. This difference may be the difference in extraction methods and solvents. The bee pollen in this study was extracted with ethanol, while it was first extracted with ethanol followed by ethyl acetate in the previous studies. A study analyzed the tyrosinase inhibitory properties of 14 types of single-flower bee pollens from China. The results showed that almost all the samples had different tyrosinase inhibitory activities due to the plant sources of bee pollen and extraction solvents. The apricot, camellia, and sunflower extracts showed excellent tyrosinase inhibitory activity [47]. In this study, the five EEBPs inhibited melanin synthesis in the mouse B16F10 melanoma cells within the concentration ranges that did not affect cellular activity. Some studies have also shown that the phenolic substances extracted from rapeseed bee pollen inhibited tyrosinase activity, thereby inhibiting melanin production [48]. DIA quantitative proteomics was used to detect the in vitro inhibitory effect of sunflower EEBP on melanin production in B16F10 cells. DEPs STAT1, caspase-3, FASN, and METTL3 were significantly up-regulated, and Heme oxygenase 1 (HO-1), Cytochrome c, and Melanophilin (MLPH) were significantly down-regulated. They were enriched in pathways related to melanin production.

The protein STAT1 usually exists in the form of a functional dimer. When STAT1 is up-regulated, it may be closely related to tumorigenesis. The biological forms of STAT1 include activated phosphorylated forms and the less active non-phosphorylated form [53]. The expression of phosphorylation STAT1 increased when normal human melanocytes (NHMs) [54] and B16F10 cells [55] were treated with the cytokine IFN-γ. In this study, expression of STAT1 in B16F10 cells was also significantly increased. Sunflower EEBP inhibits melanin production through the up-regulation of STAT1.

Caspase-3 is a cysteine–aspartic protease commonly involved in pyroptosis and apoptosis [56], but it is also involved in the processing and production of melanin. Rhododendrol inhibits tyrosinase with a decrease in melanin synthesis in mouse B16 melanoma cells and an increase in caspase-3 levels [57]. In the study of epidermal functional melanocytes and melanin loss, caffeic acid derivatives down-regulated caspase-3 in PIG1 cells, thereby protecting against melanin loss [58]. In this study, the caspase-3 protein was up-regulated after EEBP acted on B16F10 cells. Sunflower EEBP destroyed the protection of melanin loss, thereby inhibiting melanin production.

Fatty acid synthase (FASN) is an important protein that can synthesize fatty acids in cells [59]. During this process, the overexpression of METTL3 can increase the expression of FASN [60]. Different types of fatty acids can inhibit melanin production [61,62,63,64]. Both FASN and METTL3 were significantly up-regulated in this study. Sunflower EEBP increased the synthesis of fatty acids in cells and further inhibited melanin production.

Heme oxygenase 1 (HO-1) is a ubiquitous protein in most human tissues and is involved in many physiological processes [65]. Kaempferol can promote melanin synthesis and the expression of HO-1 in PIG1 cells [66] and reduce the production of ROS in the cells and the damage caused by H_2_O_2_. HO-1 was significantly down-regulated in B16F10 cells treated with EEBP. Sunflower EEBP decreased HO-1 levels and blocked the melanin synthesis pathway.

Cytochrome c is an intermembrane mitochondrial protein that interacts in the peroxidation process of various molecules. Cytochrome c can oxidize catecholamines and their S-cysteinyl derivatives, producing melanin as the final product [67]. Cytochrome c was down-regulated by the sunflower EEBP thereby reducing the synthesis of the final product melanin.

Melanophilin (MLPH) is a protein involved in the formation and transfer of melanin [68]. MLPH expression in B16F10 cells was significantly reduced after wogonin treatment [69], consistent with the down-regulation of MLPH in this study when B16F10 cells were treated with the sunflower EEBP.

## 4. Materials and Methods

### 4.1. Samples and Chemical Reagents

Rapeseed bee pollen, apricot bee pollen, camellia bee pollen, lotus bee pollen, and sunflower bee pollen were produced in Luoping County of Yunnan Province, Qinglong County of Hebei Province, Fuzhou City of Fujian Province, Jianyang County of Fujian Province, and Xuchang County of Henan Province, China, respectively. All bee pollen samples were harvested in 2023 and stored at −30 °C. The identification of the botanical origins and purities of bee pollen samples was determined by counting the percent of the matching types of pollen grains using a conventional inverted microscope (Nikon TS-100f, Tokyo, Japan) [3]. The purities of bee pollen samples are shown in Table S1. The morphologies of pollen gains are shown in Figure S1.

Dulbecco’s modified Eagle’s medium (DMEM) with high glucose, double antibiotics, and serum-free non-programmed cell freezing solution were purchased from Wuhan Pricella Biotechnology Co., Ltd. (Wuhan, China). LC-MS-grade methanol and formic acid were purchased from Thermo Fisher Scientific (Waltham, MA, USA). LC-MS-grade water was purchased from Merck, Germany. Analytical grade anhydrous ethanol and ferric chloride were purchased from Aladdin Scientific (Riverside, CA, USA). Trolox, 1,1-diphenyl-2-trinitrophenylhydrazine (DPPH), 2,2-azino-bis(3-ethyl-benzothiazole-6-sulfonic acid) diammonium salt (ABTS), petroleum ether, 2,4,6-tripyridyltriazine (TPTZ), PBS powder, mushroom tyrosinase, kojic acid, anhydrous ethanol, and sodium acetate were purchased from Shanghai Macklin Biochemical Technology Co., Ltd. (Shanghai, China). Acetic acid, PBS (pH 7.2–7.4), and hydrochloric acid were purchased from Sinopharm (Beijing, China). Trypan blue dye (0.4%) was purchased from Beijing Solarbio Science & Technology Co., Ltd. (Beijing, China). The CCK-8 kit was purchased from DOJINDO (Kumamoto, Japan). Trypsin was purchased from Hyclone Co., Logan, UT, USA. The B16F10 cell line was purchased from the China Center for Type Culture Collection (Wuhan, China).

### 4.2. Methods

#### 4.2.1. Extraction of Bee Pollen

EEBPs were prepared according to reference [52], with some modifications. Bee pollen (50 g) was crushed. The lipids of the powder were removed with 100 mL of petroleum ether at 25 °C for 30 min with ultrasonic assistance (40 kHz) two times. Then, the residue was extracted with 200 mL of 80% ethanol at 25 °C for 30 min with ultrasonic assistance two times. Subsequently, the mixture was concentrated using a rotary evaporator after filtration. It was then dried using a vacuum freeze dryer (DGJ-500H, Shanghai Boden Biotechnology Co., Ltd., Shanghai, China) to obtain the EEBP, which was then stored at −30 °C (Haier Biomedical, Qingdao, China) for further experiments.

#### 4.2.2. Determination of the Components in the Ethanol Extracts of Bee Pollens

The total phenolic contents (TPCs) of EEBPs were determined by the Folin–Ciocalteu (FC) colorimetric method [70]. Briefly, the EEBP was dissolved in ethanol and diluted to a concentration of 500 μg/mL. Then, 0.5 mL of the EEBP solution was mixed thoroughly with 2.5 mL of a 10% Folin phenol reagent in a 10 mL centrifuge tube. After 5 min, 2 mL of a 7.5% sodium carbonate solution (Na_2_CO_3_) was added to the mixture. The tube was placed in the dark at 25 °C for 60 min. The absorbance at 765 nm was measured by an ultraviolet spectrophotometer (T6, Beijing Puxi General Instrument Co., Ltd., Beijing, China). Gallic acid solutions (0.5 mL) of 10, 20, 40, 80, and 160 μg/mL were employed to establish a standard curve. The TPC is expressed as the gallic acid equivalent (mg GAE/g) contained in the EEBP.

The total flavone contents (TFCs) of EEBPs were determined using rutin standards and the NaNO_2_-Al(NO_3_)_3_-NaOH colorimetric method [43]. The EEBP was dissolved in anhydrous ethanol and diluted to 500 μg/mL. The EEBP solution (1 mL) was added to a 10 mL centrifuge tube, 0.25 mL of a 5% NaNO_2_ solution was added to mix and react for 6 min, then 0.25 mL of a 10% Al(NO_3_)_3_ solution was added to mix and react for 6 min, and finally, 2 mL of a 4% NaOH solution and 1.5 mL of ethanol were added. After standing for 15 min, the absorbance was measured at 510 nm. Rutin working solutions (1 mL) at 10, 20, 40, 80, and 160 μg/mL were used to establish the standard curve. The total flavonoid content is expressed as the rutin equivalent (mg RE/g) contained in the EEBP.

The chemical components of EEBPs were determined using a UHPLC-MS/MS system (Vanquish UHPLC system (Thermo Fisher Scientific Inc., Germering, Germany) coupled with an Orbitrap Q ExactiveTM HF-X mass spectrometer (ThermoFisher, Germering, Germany) by an untargeted metabolomic method performed by Novogene Co., Ltd. (Beijing, China) [71]. EEBP (25 mg) and 5000 μL of an 80% methanol aqueous solution were added to a 10 mL EP tube. It was whirled and centrifuged at 15,000× *g* at 4 °C for 20 min. A certain amount of the supernatant was diluted with mass spectrometry-grade water to a methanol content of 53%. It was centrifuged again for the UHPLC-MS/MS analysis. The chromatographic column was a Hypesil Gold column (C18) at 40 °C. Solutions of 0.1% formic acid and methanol were mobile phases A and B, respectively. The chromatographic gradient elution program was 0–1.5 min, 98% A; 3 min 15% A; 10 min 0% A; and 10.1–12 min 2% A at 0.2 mL/min. The mass spectrometry conditions were as follows: scan range of 100–1500 *m*/*z*; ESI source spray voltage of 3.5 kV; sheath gas flow rate of 35 psi; aux gas flow rate of 10 L/min; capillary temperature of 320 °C; s-lens RF level of 60; aux gas heater temperature of 350 °C; positive and negative polarity; and data-dependent MS/MS secondary scans.

#### 4.2.3. Antioxidant and Tyrosinase Inhibitory Capacities of Ethanol Extracts of Bee Pollens

The DPPH radical scavenging activity, FRAP total antioxidant capacity, and ABTS cation radical scavenging activity of EEBPs were determined at 517, 593, and 734 nm using an ultraviolet spectrophotometer [72]. The results are shown in Trolox equivalents (TE), μg TE/mg EEBP.

The tyrosinase inhibitory capacities were determined according to reference [3], with some modifications. EEBPs were dissolved in ethanol (2 mg/mL) and then diluted to different concentrations (10–50 μg/mL) with PBS buffer (pH 6.8). A sample solution of 25 μL and 25 μL of tyrosinase solution (200 U/mL) were added to a 96-well plate and pre-incubated at 37 °C for 2 min, and then 200 μL of L-DOPA (0.5 mM) was added to each well for the reaction. The absorbance of the mixture was measured at 475 nm every 20 s at 37 °C for a total of 20 times. The inhibition rate was calculated as follows:

Inhibition rate (%) = (1 − k1/k0) × 100(1)

where k1 is the slope of the kinetic equation of the sample group and the positive control group, and k0 is the slope of the kinetic equation of the blank control group.

#### 4.2.4. Anti-Melanogenesis Activities of Ethanol Extracts of Bee Pollens on Melanin Production in Mouse B16F10 Melanoma Cells 

Mouse B16F10 melanoma cells were cultured in high-glucose DMEM (containing 10% FBS fetal bovine serum and 1% double antibiotics) in a 5% CO_2_ incubator at 37 °C (C150, Binder, Tuttlingen, Germany). The cell number in suspension (100 μL) was counted with the assistance of trypan blue staining (100 μL). The concentration of cells was diluted to 5 × 10^4^ cells/mL with the culture medium. Add 100 μL of cell suspension to each well of the 96-well plate. These cells were cultured for 24 h. The culture medium was removed and 100 μL of sterile PBS was added to each well for washing. Culture medium (100 μL) containing 5, 10, 15, 20, or 25 μg/mL EEBPs were added to 6 wells for each dose. Wells containing whole culture medium were the control group. Cells were continuously cultured in the incubator for 48 h. These cells were washed with sterile PBS. And then, 110 μL of culture medium containing the CCK-8 solution was added in a dark place. The cells were cultured for 2 h. Then, the absorbance at 450 nm was measured.

Cell proliferation activity (%) = [(OD of the experimental group − OD of the blank group)/(OD of the control group − OD of the blank group)] × 100(2)

The determination of the anti-melanogenesis effect of EEBPs was performed according to the method of Sim et al. [73], with slight changes. Cells at a concentration of 1 × 10^4^ cells/mL were transferred into 6-well plates. After treatment with 5, 10, 15, 20, or 25 μg/mL EEBP for 48 h, the cells in each group were collected into centrifuge tubes and counted. After centrifugation, 0.5 mL of a 1 mM NaOH solution (containing 10% DMSO) was added and heated in a water bath at 80 °C for 2 h. The absorbance at 405 nm was measured using an ELISA reader.

Relative melanin content (%) = (OD of experimental group/number of cells in the experimental group)/(OD of blank group/number of blank cells) × 100(3)

The correlations among DPPH, FRAP, ABTS, TPC, TFC, antityrosinase, and anti-melanogenesis activities were analyzed.

#### 4.2.5. Analysis of the Anti-Melanogenesis Mechanism of the Ethanol Extract of Sunflower Bee Pollen on Melanin Production in Mouse B16F10 Melanoma Cells by Label-Free Proteomics

Differentially expressed proteins in mouse melanoma cells (B16F10) treated with sunflower EEBP, compared to a control group, were determined using DIA quantitative proteomics. In brief, 2 mL of cells at a concentration of 1 × 10^5^ cells/mL were added to a 6-well plate and cultured for 24 h. After removing the culture medium, the cells were rewashed with 2 mL of PBS (pH 7.2–7.4). The cells were then incubated with 2 mL of culture medium containing 25 μg/mL sunflower EEBP (resulting in a final concentration of 25 μg/mL) for 48 h. Following this, the cells were washed twice with pre-cooled PBS at 4 °C and then digested with trypsin (0.25%). The collected cells were washed again with pre-cooled PBS and centrifuged (TDZ4K, Hunan Xiangyi Laboratory Instrument Development Co., Ltd., Changsha, China) at 137× *g* for 5 min twice. The cells were frozen in liquid nitrogen for 15 min and stored at −80 °C in a Haier Biomedical refrigerator (Qingdao, China). The extraction and determination of the total protein concentration, as well as the protein spectral analysis, were performed as outlined in a previous report [71].

#### 4.2.6. Statistical Analysis

All experiments except for the chemical substance determination were performed in triplicate. Except for the proteomic experiment, one-way ANOVA was used to analyze the differences using GraphPad Prism 8.4.3 for Windows (GraphPad Software, Inc. San Diego, CA, USA), where *p* < 0.01 means an extremely statistically significant difference between the treatment group and the control group, and *p* < 0.05 means a statistically significant difference. The results are expressed as means ± standard errors.

The offline data files for the chemical substance determination were imported into CD 3.3 library search software for processing. Each metabolite was screened by parameters such as the retention time and mass-to-charge ratio. Then, the peak area was corrected with the first QC to make the identification more accurate. Then, the mass deviation was set to 5 ppm, the signal intensity deviation was 30%, and the minimum signal intensity, the adduct ion, and other information were set for peak extraction. At the same time, the peak area was quantified, and the target ion was integrated. The molecular formula was predicted by the molecular ion peak and fragment ion and compared with the mzCloud (https://www.mzcloud.org/, accessed on 10 August 2024), mzVault, and Masslist databases. A blank sample was employed to remove the background ions, and the original quantitative results were standardized to obtain the relative peak area. The compounds with a relative peak area CV higher than 30% in the QC sample were deleted, and, finally, the metabolite identification and relative quantitative values were obtained using the following formula: sample metabolite original quantitative value/(sum of sample metabolite quantitative values/sum of QC1 sample metabolite quantitative values). The data processing was based on the Linux operating system (CentOS v6.6) and R (R-3.4.3) and Python (Python-3.5.0) software.

The spectra obtained from LC-MS/MS were analyzed as described in a previous report [74]. The proteins whose quantities were significantly different between the experimental and control groups (*p* ≤ 0.05 and fold change (FC) > 1.2 or FC < 0.83) were defined as differentially expressed proteins (DEPs).

## 5. Conclusions

Each type of bee pollen has unique substances. The antityrosinase activity of sunflower EEBP and anti-melanogenesis activity in B16F10 cells were the highest among the rapeseed, apricot, camellia, lotus, and sunflower EEBPs. The anti-melanogenesis activities of bee pollens were positively correlated with their antityrosinase activities and total phenol contents. The sunflower EEBP inhibited melanogenesis in B16F10 cells by reducing the expression of related proteins in the cAMP, MAPK, and TNF signaling pathways. This result provides a basis for the future industrial development of a raw material for skin whitening using sunflower bee pollen.

## Figures and Tables

**Table 1:** Total phenolic and flavonoid contents of ethanol extracts of bee pollens.

Bee Pollen	TPC (mg GAE/g)	TFC (mg RE/g)
Rapeseed	64.80 ± 1.34 ^c^	79.81 ± 1.62 ^b^
Apricot	79.00 ± 1.14 ^b^	66.55 ± 4.72 ^c^
Camellia	64.29 ± 1.64 ^c^	64.1 9± 1.77 ^c^
Lotus	5.36 ± 0.50 ^d^	1.99 ± 0.45 ^d^
Sunflower	132.57 ± 1.41 ^a^	109.06 ± 1.35 ^a^

Different lowercase characters in the same column mean statistically significant differences (*p* < 0.05).

**Table 2:** Flavonoids in ethanol extracts of bee pollens.

Name	Formula	Molecular Weight	Retention Time (min)	*m*/*z*	Relative Quantitative Value ^#^					Polarity Mode
					A	B	C	D	E	
4′,7-Dihydroxyflavanone	C_15_ H_12_ O_4_	256.07342	6.011	257.0807	ND *	421,160,345.9	2,761,299.049	2,878,041.662	ND	pos
Paracetamol	C_8_ H_9_ N O_2_	151.06328	5.696	152.0706	43,034,369.2	76,811,021.73	376,365,295.8	147,656,556.3	388,854,376.3	pos
Diosmetin	C_16_ H_12_ O_6_	300.06318	6.039	301.0705	214,452,989	191,074,539.2	52,141,965.55	24,221,989	32,956,884.01	pos
Galangin	C_15_ H_10_ O_5_	270.05258	6.642	271.0599	71,935,041.1	78,118,724.65	39,386,575.66	20,616,903.96	ND	pos
Isorhamnetin	C_16_ H_12_ O_7_	316.0581	5.561	317.0654	684,415,707	2,700,227,153	848,073,257	1,339,085,340	410,416,525.5	pos
Kaempferol	C_15_ H_10_ O_6_	286.04762	5.783	287.0549	421,485,230	542,513,047.8	1,083,001,678	3,746,952,740	4,545,692,793	pos
Apigenin	C_15_ H_10_ O_5_	270.05266	6.035	271.0599	59,073,999	492,996,321.1	34,174,186.03	103,121,637.5	15,609,215.28	pos
Chrysin	C_15_ H_10_ O_4_	254.05766	6.584	255.0649	386,526,326	472,417,967.1	187,207,714.9	146,964,633.5	25,581,440.96	pos
Hesperetin	C_16_ H_14_ O_6_	302.07906	5.396	303.0863	ND	1,206,039,601	5,768,035.731	70,706,093.49	19,793,290.76	pos
4-Ethylphenol	C_8_ H_10_ O	122.07331	7.007	123.0805	232,956,283	219,609,736.9	581,174,572.1	848,615,029.3	744,324,047.7	pos
3-Hydroxyphenylacetic acid	C_8_ H_8_ O_3_	152.04652	3.448	153.0538	1,258,417,229	495,499,205.9	32,153,838.89	178,078,042.9	156,571,841.1	pos
DL-Metanephrine	C_10_ H_15_ N O_3_	197.1052	1.438	198.1125	44,608,165.2	14,591,930.83	25,372,108.46	174,820,069.1	493,116,291	pos
Robinetin	C_15_ H_10_ O_7_	132.04349	5.472	303.0501	7,228,439.57	192,17,361,011	347,711,367.3	295,845,940.9	113,126,228.6	pos
Isoeugenol	C_10_ H_12_ O_2_	164.08379	5.872	165.0911	121,446,929	168,376,512.1	507,361,781.8	664,056,676.7	257,748,794.5	pos
N-Oleoyl dopamine	C_26_ H_43_ N O_3_	439.30561	7.476	440.3129	ND	186,795,049.6	ND	7,434,264.734	ND	pos
4′-O-Glucosylvitexin	C_27_ H_30_ O_15_	594.15738	5.303	595.1647	137,988,745	201,390,496.8	12,019,090.94	123,593,071.6	21,336,459.52	pos
Epicatechin	C_15_ H_14_ O_6_	308.08945	5.238	291.0861	ND	ND	ND	158,962,728.2	ND	pos
Dihydrocapsaicin	C_18_ H_29_ N O_3_	307.21458	5.781	308.2219	13,025,748.8	5,255,758.773	28,698,096.6	33,645,843.33	58,189,390.76	pos
Vanillin	C_8_ H_8_ O_3_	170.05787	5.896	188.0917	18,507,044.3	39,733,515.43	23,754,186	16,790,489.85	23,462,102.25	pos
Antiarol	C_9_ H_12_ O_4_	184.07359	5.897	185.0811	ND	34,692,677.88	33,181,916.55	11,779,943.87	13,883,890.37	pos
Rhoifolin	C_27_ H_30_ O_14_	616.11857	11.184	617.1259	ND	ND	ND	83,619,032.81	115,639,570.7	pos
Narcissoside	C_28_ H_32_ O_16_	624.17131	5.543	625.1786	12,927,457.8	6,672,526.705	3,798,041.678	4,862,624.146	ND	pos
Neodiosmin	C_28_ H_32_ O_15_	646.12896	10.452	647.1362	10,404,117.8	7,699,224.206	ND	227,282,528.5	222,436,152.3	pos
(+)-Catechin	C_15_ H_14_ O_6_	290.0791	5.095	291.0864	ND	1,884,259.734	ND	62,231,803.58	ND	pos
Isovanillin	C_8_ H_8_ O_3_	152.04737	4.003	170.0812	43,279,880.4	36,658,145.71	57,981,490.76	49,821,480.76	43,032,161.47	pos
Olivetol	C_11_ H_16_ O_2_	180.11491	6.118	181.1222	1,579,855,301	219,826,697.2	1,867,879,237	7,178,979,570	4,250,002,794	pos
N-Sinapoylputrescine	C_15_ H_22_ N_2_ O_4_	294.15804	5.245	295.1653	223,796,341	85,396,936	2,034,501,055	114,756,689.9	642,065,079.6	pos
m-Cresol	C_7_ H_8_ O	108.05771	6.987	109.065	453,056,628	492,797,783.9	760,132,605.4	934,468,079.3	1,352,699,960	pos
N-Feruloylspermidine	C_17_ H_27_ N_3_ O_3_	321.20493	5.311	322.2122	16,227,369.5	ND	ND	388,149,239	291,830,830.8	pos
Syringetin	C_17_ H_14_ O_8_	346.06869	5.989	347.076	213,797,430	36,006,183.47	83,525,509.69	56,476,876.03	2,790,118.961	pos
10-Gingerol	C_21_ H_34_ O_4_	350.24526	6.449	351.2525	63,159,023.2	31,907,273.33	85,237,129.4	59,818,272.11	185,615,026	pos
Synephrine	C_9_ H_13_ N O_2_	167.09488	5.367	168.102	11,473,821.2	10,736,209.71	32,505,178.53	29,179,639.69	37,779,114.95	pos
Homovanillic acid	C_9_ H_10_ O_4_	182.0578	6.121	165.0545	36,791,534.7	53,843,338.18	98,234,762.32	79,103,854.53	64,102,700.09	pos
3-Hydroxy-glabrol	C_25_ H_28_ O_5_	408.19311	2.638	409.2004	15,627,920.5	7,577,848.342	12,982,293.77	2,144,060.696	65,127,151.99	pos
Eupatilin	C_18_ H_16_ O_7_	344.08942	6.993	345.0967	ND	55,836,940	28,821,311.22	ND	ND	pos
Cyanin chloride	C_27_ H_31_ Cl O_16_	646.12943	7.508	647.1367	ND	ND	ND	80,724,006.14	78,662,587.44	pos
N-Caffeoylagmatine	C_14_ H_20_ N_4_ O_3_	292.15315	5.011	585.3145	7,508,559.99	ND	24,444,379.22	ND	31,458,991.44	pos
Capsaicin	C_18_ H_27_ N O_3_	305.19874	6.452	306.206	12,087,681.5	2,776,542.382	25,089,124.12	17,821,873.15	15,588,985.07	pos
(−)-Epigallocatechin	C_15_ H_14_ O_7_	306.07351	5.599	307.0808	ND	ND	ND	53,832,441.21	13,548,177.99	pos
Tangeretin	C_20_ H_20_ O_7_	372.12075	5.423	355.1177	ND	ND	ND	5,917,055.075	ND	pos
Tyrosol	C_8_ H_10_ O_2_	138.06757	5.138	299.1232	ND	5,068,007.118	6,256,197.387	3,557,810.413	5,186,575.09	pos
Epigallocatechin gallate	C_22_ H_18_ O_11_	458.0842	5.285	457.0769	ND	ND	ND	33,786,926.33	2,234,317.434	neg
Luteolin	C_15_ H_10_ O_6_	286.04732	10.293	285.04	40,846,755.4	35,743,531.6	65,613,907.63	22,530,517.56	33,920,595.23	neg
Quercetin-3β-D-glucoside	C_21_ H_20_ O_12_	464.09462	5.497	463.0873	7,811,562.25	31,223,348,577	424,844,619.2	93,067,914.68	49,376,059.55	neg
Quercetin	C_15_ H_10_ O_7_	302.04189	6.168	301.0346	50,861,607.5	3,456,250,485	167,546,683.1	100,938,577	9,389,005.75	neg
Naringenin	C_15_ H_12_ O_5_	272.06788	5.811	271.0606	696,497,190	2,964,782,677	26,471,899.02	157,562,617.8	15,620,342.86	neg
Trifolin	C_21_ H_20_ O_11_	448.09963	5.586	447.0924	15,251,810.2	3,616,379,862	219,125,099.9	768,635,754.3	672,018,938.1	neg
Myricetin	C_15_ H_10_ O_8_	318.03709	11.206	317.0298	ND	3,028,274.129	ND	19,796,260.72	8,338,753.585	neg
Catechin	C_15_ H_14_ O_6_	290.07854	5.233	289.0713	ND	11,479,131.28	ND	362,680,090	21,989,649.4	neg
Rutin	C_27_ H_30_ O_16_	610.15255	5.406	609.1453	4,896,680,579	11,032,993,617	4,297,271,375	391,130,440.3	181,767,093.3	neg
Camelliaside A	C_33_ H_40_ O_20_	756.21056	5.419	755.2033	1,856,076,137	152,399,114.1	9,105,591.564	4,331,242,550	2,044,379,996	neg
Vitexin	C_21_ H_20_ O_10_	432.10434	5.582	477.1025	25,549,827.3	3,047,473,762	142,276,104.5	693,348,490.6	225,742,072.2	neg
Quercetin-3-O-beta-glucopyranosyl-6′-acetate	C_23_ H_22_ O_13_	506.10501	5.508	505.0977	3,746,894.48	1,951,523,698	22,777,582.34	5,963,197.709	ND	neg
(−)-Gallocatechin	C_15_ H_14_ O_7_	306.07093	1.48	305.0636	426,513,401	202,996,217.7	352,989,760.4	778,605,608.9	197,179,065.1	neg
C-pentosyl-apeignin O-feruloylhexoside	C_36_ H_36_ O_17_	740.19445	5.658	739.1872	ND	ND	4,277,967.468	482,030,561.8	398,168,515.7	neg
Lysionotin	C_18_ H_16_ O_7_	344.08877	6.465	343.0815	5,045,809.68	366,547,540.5	ND	ND	ND	neg
Morin Hydrate	C_15_ H_12_ O_8_	320.05248	5.669	319.0452	40,211,091.5	ND	ND	9,452,094.08	6,808,868.601	neg
Laricitrin	C_16_ H_12_ O_8_	332.05252	5.702	331.0452	30,519,617	24,396,606.59	15,799,130.1	313,192,808.2	5,637,949.068	neg
Liquiritigenin	C_15_ H_12_ O_4_	256.07285	5.724	255.0656	ND	222,165,135.5	ND	ND	ND	neg
Scutellarin	C_21_ H_18_ O_12_	462.07957	5.554	461.0723	ND	5,717,791.241	264,675,766.2	ND	ND	neg
Astragaloside I	C_45_ H_72_ O_16_	868.48566	9.474	867.4784	192,740,828	ND	9,275,827.199	ND	ND	neg
Procyanidin B2	C_30_ H_26_ O_12_	578.1422	5.083	577.1349	ND	ND	ND	148,408,865.7	5,040,187.901	neg
Dihydromyricetin	C_15_ H_12_ O_8_	320.05263	5.306	319.0454	ND	ND	ND	85,023,185.27	2,195,644.614	neg
Typhaneoside	C_34_ H_42_ O_20_	770.21135	5.284	769.2041	159,063,911	1,31,733,637.6	34,066,220.05	231,660,164.2	63,182,732.09	neg
Flavanone	C_15_ H_12_ O_2_	224.08876	3.351	223.0815	20,038,004.3	1,65,971,467.7	9,437,481.559	19,717,896.64	8,643,483.933	neg
Epicatechin-3-O-gallate	C_22_ H_18_ O_10_	442.08902	5.363	441.0817	ND	ND	ND	42,171,533.16	3,743,314.08	neg
Theaflavin	C_29_ H_24_ O_12_	564.12608	5.844	563.1188	ND	ND	ND	28,600,078.21	33,086,570.62	neg
Casticin	C_19_ H_18_ O_8_	374.09987	6.383	373.0926	ND	26,391,447.21	ND	ND	ND	neg
Tricin O-malonylhexoside	C_26_ H_26_ O_15_	578.12501	5.27	577.1177	ND	29,288,856.87	ND	ND	ND	neg
Wogonoside	C_22_ H_20_ O_11_	460.09926	5.694	459.092	ND	ND	ND	17,955,043.79	13,013,533.49	neg
Methyl Hesperidin	C_29_ H_36_ O_16_	640.20009	5.25	639.1928	ND	ND	ND	7,345,122.487	ND	neg
Isosilybin	C_25_ H_22_ O_10_	482.12057	5.762	481.1133	ND	ND	18,684,411.97	ND	ND	neg
Engeletin	C_21_ H_22_ O_10_	434.12086	5.569	433.1136	ND	13,637,214.15	10,381,819.84	ND	ND	neg
Heptamethoxyflavone	C_22_ H_24_ O_9_	432.14137	5.495	431.1341	ND	ND	7,644,360.736	ND	ND	neg
Isovitexin	C_21_ H_20_ O_10_	432.10562	6.007	431.0983	ND	ND	3,278,357.703	ND	ND	neg
Pelargonidin chloride	C_15_ H_11_ Cl O_5_	306.02892	6.223	305.0217	ND	ND	ND	ND	ND	neg
Pectolinarin	C_29_ H_34_ O_15_	622.1918	10.469	621.1845	1,634,093.99	3,236,392.594	1,918,622.412	ND	981,732.339	neg
Astilbin	C_21_ H_22_ O_11_	450.11592	4.901	449.1086	ND	ND	ND	3,294,421.57	ND	neg
Cyanidin chloride	C_15_ H_11_ Cl O_6_	322.0258	5.564	321.0185	ND	ND	2,971,429.103	ND	ND	neg
(−)-Catechin Gallate	C_22_ H_18_ O_10_	442.08978	4.971	423.0719	ND	ND	ND	3,956,997.454	ND	neg
6-Shogaol	C_17_ H_24_ O_3_	276.17204	7.294	275.1649	ND	ND	1,419,321.159	ND	ND	neg
Orientin	C_21_ H_20_ O_11_	448.10042	4.997	447.0931	ND	ND	ND	ND	ND	neg
Baohuoside I	C_27_ H_30_ O_10_	514.18345	1.851	513.1762	ND	ND	479,069.6341	ND	ND	neg
Tulipanin	C_27_ H_31_ O_16_	611.16334	4.729	610.1561	ND	ND	ND	1,155,753.351	807,483.1285	neg
Kuwanon A	C_25_ H_24_ O_6_	420.1573	1.995	419.15	ND	124,712.2319	ND	154,082.8984	ND	neg
Vitexin-2-O-rhaMnoside	C_27_ H_30_ O_14_	578.16401	2.963	577.1567	ND	ND	ND	341,012.0014	245,379.7201	neg
Quercetin 3-O-sophoroside	C_27_ H_30_ O_17_	626.14729	5.308	625.14	12,969,050	5,464,610,685	22,465,559.36	385,733,174.2	62,948,432.34	neg
Sinapyl Alcohol	C_11_ H_14_ O_4_	210.08836	5.613	209.0811	ND	ND	3,982,772.239	58,595,380.89	93,954,803.85	neg
Pyrogallol	C_6_ H_6_ O_3_	126.03056	3.136	125.0233	52,253,814.5	50,876,134.5	54,751,955.84	5,056,863,601	4,650,663,073	neg
Cyanidin 3-rutinoside	C_27_ H_31_ O_15_	595.16789	4.9	594.1606	ND	ND	ND	78,888,627.46	3,570,249.836	neg
Eriodictyol	C_15_ H_12_ O_6_	288.06298	6.017	269.0451	37,755,502.9	151,216,709.3	18,272,926.02	41,340,090.1	4,827,039.11	neg
Vaccarin	C_32_ H_38_ O_19_	726.19982	5.289	725.1928	ND	63,646,196.73	ND	ND	5,883,643.504	neg
6-Paradol	C_17_ H_26_ O_3_	278.1878	5.89	323.186	185,053,697	305,250,229.3	295,294,310.1	275,009,885.5	659,984,250.6	neg
Epigallocatechin	C_15_ H_14_ O_7_	306.07363	4.832	305.0664	ND	ND	ND	37,577,124.11	3,325,753.027	neg
Taxifolin	C_15_ H_12_ O_7_	304.05811	5.441	303.0505	3,570,091.41	16,824,267.62	21,717,034.58	7,035,144.266	2,900,749.473	neg
Quercetin 3-alpha-L-arabinofuranoside (Avicularin)	C_20_ H_18_ O_11_	434.08376	5.554	433.0765	ND	72,544,130.62	2,043,273.191	5,471,247.624	ND	neg
Ferulaldehyde	C_10_ H_10_ O_3_	178.06273	7.239	355.1183	20,749,154.8	22,025,828.83	4,208,647.71	7,550,227.058	ND	neg

* ND means that the substance was not detected in the ethanol extract of the bee pollen. A, B, C, D, and E represent rapeseed, apricot, camellia, lotus, and sunflower bee pollens, respectively. ^#^ The relative quantitative value of each component can be compared among different EEBPs, but it cannot be compared among different components.

**Table 3:** Antioxidant capacities of ethanol extracts of bee pollens.

Bee Pollen	DPPH (μg TE/mg)	FRAP (μg TE/mg)	ABTS (μg TE/mg)
Rapeseed	76.03 ± 1.17 ^b^	123.82 ± 3.79 ^b^	177.31 ± 1.39 ^b^
Apricot	31.36 ± 1.44 ^d^	56.04 ± 2.22 ^d^	174.30 ± 1.88 ^b^
Camellia	60.03 ± 1.49 ^c^	96.34 ± 2.41 ^c^	166.14 ± 2.80 ^b^
Lotus	2.50 ± 0.18 ^e^	8.29 ± 0.48 ^e^	13.80 ± 0.52 ^c^
Sunflower	119.26 ± 2.17 ^a^	156.83 ± 4.62 ^a^	204.88 ± 1.89 ^a^

Different superscript lowercase characters in the same column mean statistically significant differences (*p* < 0.05).

**Table 4:** The correlation coefficients among DPPH, FRAP, ABTS, TPC, TFC, tyrosinase inhibitory, and anti-melanogenesis activities.

	DPPH	FRAP	ABTS	TPC	TFC	Tyrosinase Inhibitory	Anti-Melanogenesis
DPPH	1						
FRAP	0.986 **	1					
ABTS	0.800	0.849	1				
TPC	0.879 *	0.885	0.849 *	1			
TFC	0.924 *	0.939 *	0.959 **	0.953 *	1		
Tyrosinase Inhibitory	0.722	0.682	0.827	0.964 *	0.866	1	
Anti-Melanogenesis	0.675	0.633	0.811	0.940 *	0.826	0.987 **	1

Characters “*” and “**” mean significant differences at *p* < 0.05 and *p* < 0.01, respectively.

**Table 5:** The differentially expressed proteins enriched in pathways highly related to melanogenesis in mouse B16F10 melanoma cells treated with the ethanol extract of sunflower bee pollen compared with the control group.

Pathway	Up-Regulated Proteins	Down-Regulated Proteins
MAPK		MAP2K1, NFKB2, RELB, RPS6KA3, CASP3, TRAF6, MAP2K5, MAPKAPK3
cAMP	ADCY7, GRIN2A	MAP2K1
AMPK	CPT1A	STRADA, CCNA2, FASN

## Data Availability

Data are contained within this article.

## Supplementary Materials

The following supporting information can be downloaded at https://www.mdpi.com/article/10.3390/ph17121634/s1: Table S1. The purity of bee pollen samples. Figure S1. Morphology of bee pollen grains. Figure S2. Volcano map of proteins in B16F10 cells (sunflower EEBP vs. control).
