# Supplementary material for: Polyphenol Profile and Antioxidant, Antityrosinase, and Anti-Melanogenesis Activities of Ethanol Extract of Bee Pollen
Source: Pharmaceuticals (Basel). 2024 Dec 5;17(12):1634. doi: 10.3390/ph17121634 (PMC11728481; doi:10.3390/ph17121634)
Supplement: Supplementary file 1 [file pharmaceuticals-17-01634-s001.zip › pharmaceuticals-3317553-supplementary.pdf]

Supplement

Table S1. The purity of bee pollen samples.

Botanical Source of Bee Pollen	Purity
Rapeseed	97.98%
Apricot	96.61%
Camellia	99.07%
Lotus	98.25%
Sunflower	93.77%

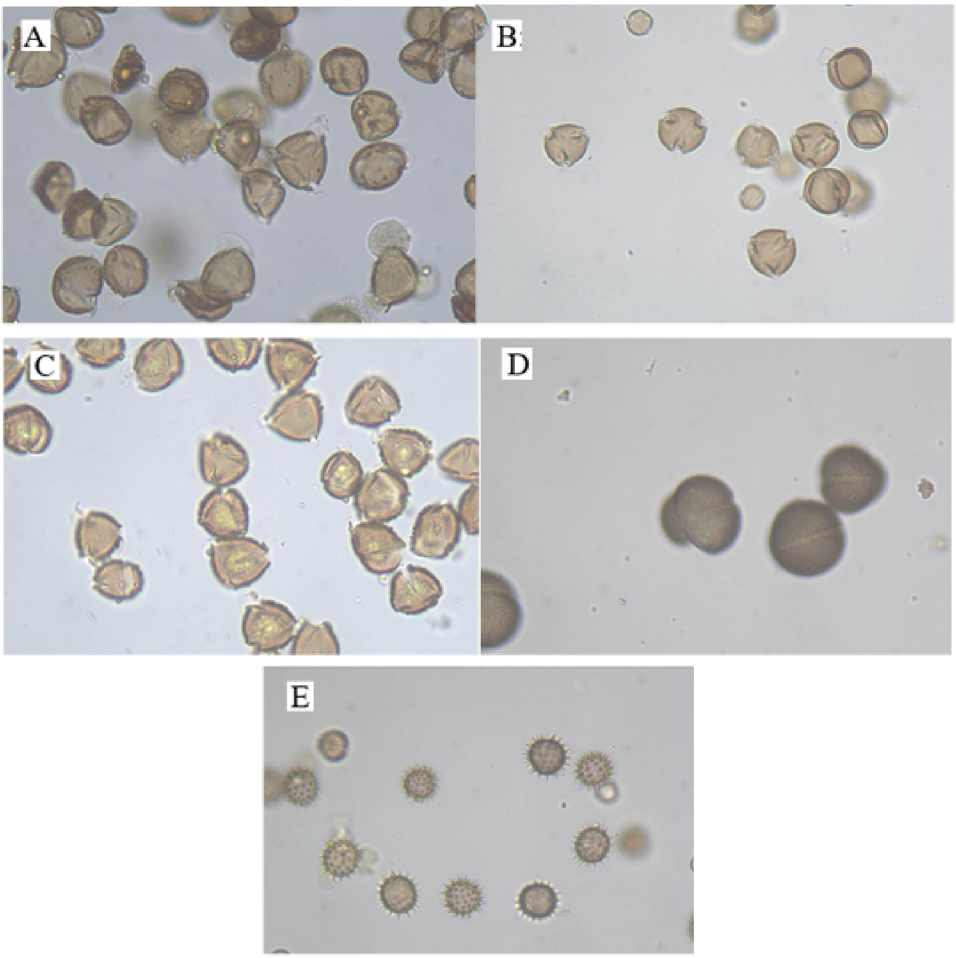

Figure S1. Morphology of bee pollen grains. Note: A. rapeseed, B. apricot, C. camellia, D. lotus, and E. sunflower.

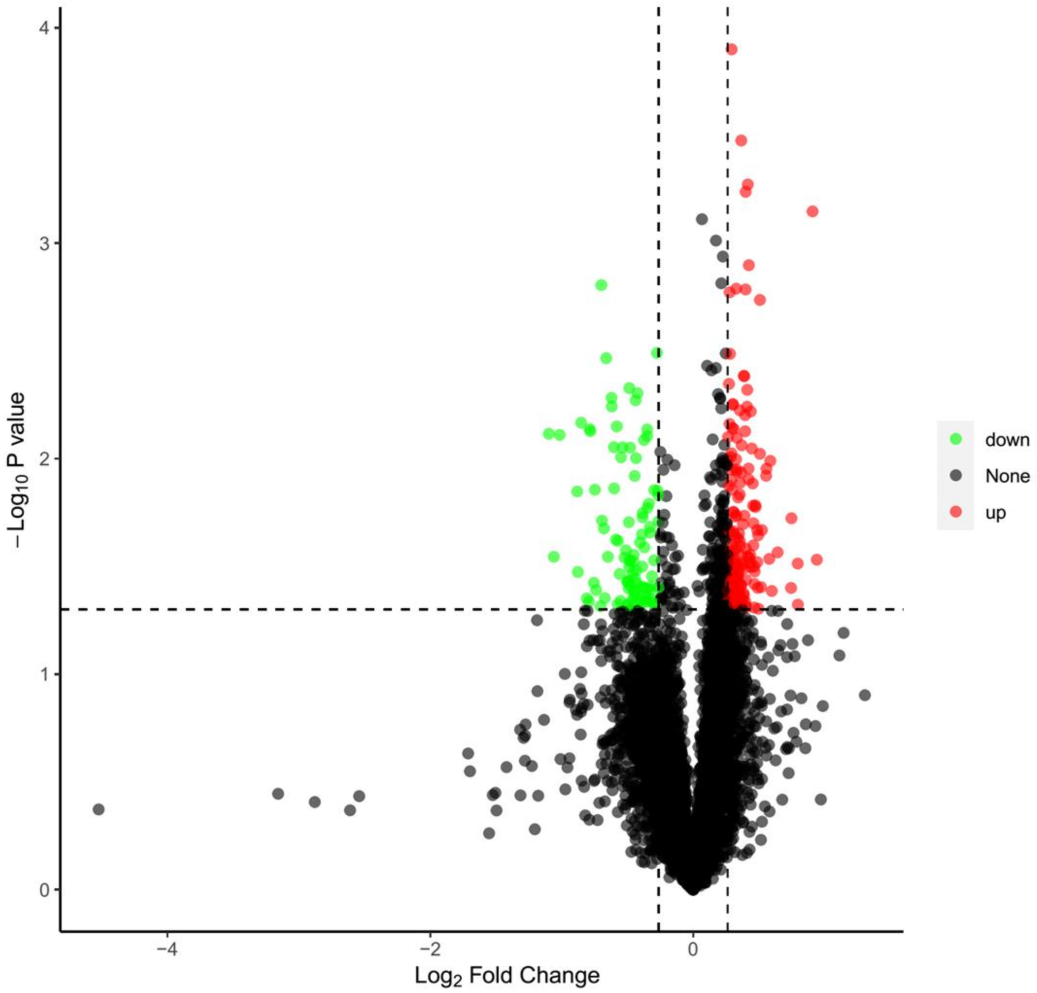

Figure S2. Volcano map of proteins in B16F10 cells (sunflower EEBP vs. control). Note: Green, red, and black dots represent down-, up-, and non-regulated proteins.
